# Blender tissue cartography: an intuitive tool for the analysis of dynamic 3D microscopy data

**DOI:** 10.1101/2025.02.04.636523

**Published:** 2025-02-08

**Authors:** Nikolas Claussen, Cécile Regis, Susan Wopat, Sebastian Streichan

**Affiliations:** 1 Department of Physics, University of California Santa Barbara, Santa Barbara, California 93106, USA; 2 Department of Bioengineering, University of California Santa Barbara, Santa Barbara, California 93106, USA

## Abstract

Tissue cartography extracts and cartographically projects surfaces from volumetric biological image data. This turns 3D- into 2D data which is much easier to visualize, analyze, and computationally process. Tissue cartography has proven particularly useful in developmental biology by taking advantage of the sheet-like organization of many biological tissues. However, existing software tools for tissue cartography are limited in the type of geometries they can handle and difficult for non-experts to use and extend. Here, we describe blender_tissue_cartography (btc), a tissue cartography add-on for the popular 3D creation software Blender. btc makes tissue cartography user-friendly via a graphical user interface and harnesses powerful algorithms from the computer graphics community for biological image analysis. The btc GUI enables interactive analysis and visualization without requiring any programming expertise, while an accompanying Python library allows expert users to create custom analysis pipelines. Both the add-on and the Python library are highly modular and fully documented, including interactive Jupyter Notebook tutorials. btc features a general-purpose pipeline for time-lapse data in which the user graphically defines a cartographic projection for a single *key frame*, which is propagated to all other frames via surface-to-surface alignment algorithms. The btc differential geometry module allows mathematically correcting for cartographic distortion, enabling faithful 3D measurements in 2D cartographic projections, including for vector fields like tissue flow fields. We demonstrate btc on diverse and complex tissue shapes from *Drosophila*, stem-cell-based organoids, *Arabidopsis*, and zebrafish.

## Introduction

Biological objects such as tissues, organs, or entire embryos and animals, are inherently three-dimensional (3D), requiring volumetric microscopy. This can make data storage, visualization, and quantitative analysis challenging. Tissue cartography takes advantage of the layered organization of many biological structures, such as epithelia, leaves, or tube-like visceral organs, to represent structures of interest as curved 2D surfaces embedded in 3D[1]. A tissue cartography workflow begins with the segmentation of a *surface of interest* (SOI) from 3D volumetric data (e.g. a *z*-stack from a confocal microscope), which is then cartographically *unwrapped* to a 2D plane. The volumetric image data can then be visualized and analyzed in the cartographic projection, reducing 3D- to 2D image analysis. This greatly facilitates tasks such as cell segmentation and tracking [2], measurement of tissue deformation [3], quantification of protein localization patterns [4], and *in-toto* visualization of curved objects. Tissue cartography has proven useful notably in developmental biology where tissues and organs dynamically change shape.

Here, we present blender_tissue_cartography (btc), an add-on to the popular, open-source 3D editor Blender [5] and an accompanying Python library. The btc suite makes the analysis of dynamic, highly curved surfaces via tissue cartography accessible to non-experts by taking advantage of high-quality tools developed by the computer graphics and 3D animation community. btc features a unified, modular design, a graphical user interface, and a general-purpose pipeline for the analysis of dynamic datasets. btc comes with detailed documentation, tutorials, and a set of template analysis pipelines.

### Prior work

Existing tools such as for tissue cartography have enabled striking, quantitative insights into 3D morphogenesis [6, 2, 7]. This highlights the utility of the method. Yet, these implementations have shortcomings that impede widespread adoption and analysis of complex, dynamic geometries.

Tools like LocalZProjector [8] parameterize surfaces via a *height function* and hence cannot handle fully 3D surfaces with “overhang”. The 3D analysis software MorphoGraphX [9] does not carry out cartographic projections at all and instead locally projects image intensity onto the vertices of a high-resolution surface mesh. This approach sidesteps cartographic projections but has certain limitations. In 3D, only part of a curved surface is visible at any time, and analysis of mesh-vertex-associated data is more complicated and less flexible than true cartographic projections, especially if the image cannot be easily segmented into cells.

Our approach is most closely related to the ImSAnE [1] and TubULAR [3] software packages. Due to a lack of a graphical interface for editing surfaces and their cartographic projection and reliance on specialized MATLAB code, however, these tools are challenging for non-experts to use. Further, ImSAnE (developed in 2015) does not take advantage of sophisticated surface-processing algorithms developed by the computer vision community over the last decade (e.g. [10, 11]), while the more recent TubULAR is specialized to tube-like surfaces only.

## Methods

### Tissue cartography workflow in btc

**Figure 1:**
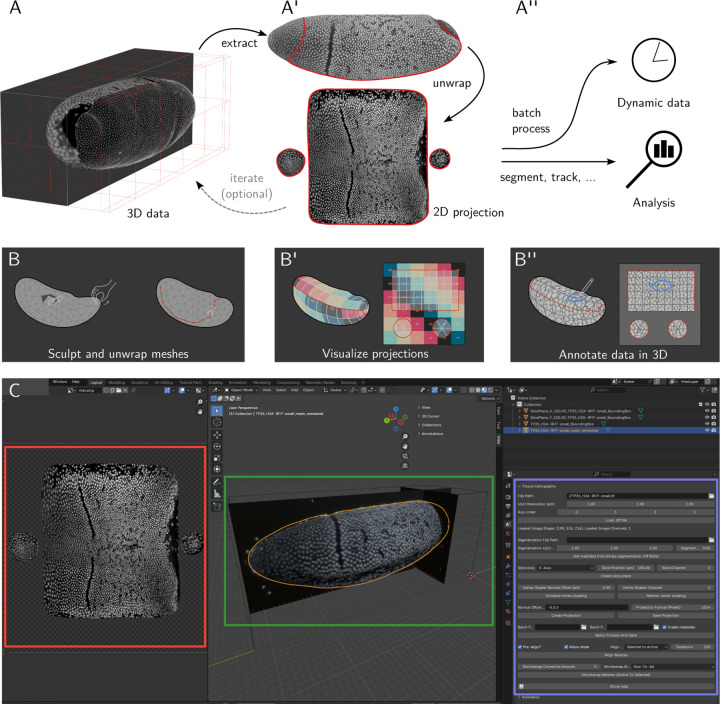
Tissue cartography workflow in btc. **A-A”** (A) 3D image data image (*Drosophila* blastoderm with fluorescently labeled nuclei, anterior left, ventral down). (A’) The extracted curved 2D surface of interest is represented by a polygonal mesh. Surface unwrapping (UV mapping) allows the projection of 3D image data into 2D. After inspection of the results, meshes and UV maps can be rapidly edited to improve the quality of 2D projections. (A”) 2D projections can be used for *in toto* visualization or downstream analyses such as cell segmentation. Batch processing tools allow the processing of dynamic datasets (see Fig. 2). Modular design allows switching out the tool used in each pipeline step. **B-B”** Interactive 3D data processing in Blender’s graphical user interface. (B) Surface sculpting for fixing segmentation errors. Blender’s UV editor combines powerful cartographic algorithms with graphical user input (e.g. “seam” placement). (B’) Cartographic projections and their distortion can be visualized in 3D, for instance by mapping a color grid onto the mesh surface. (B”) Projected data can be visualized and annotated in 3D, with results saved to 2D projections. **C** Blender user interface. Cartographic projection (red), surface of interest with 3D data visualized by ortho-slices and projected mesh intensity (green), btc add-on toolset for loading and processing 3D data (blue).

Tissue cartography generally proceeds in the following steps (Fig. 1A–A”):

1. **Extraction:** Detect the surface of interest in volumetric image data, typically via 3D image *segmentation*, and convert the result to a surface mesh (*meshing*).
2. **Unwrapping:** Unwrap the 3D surface to a 2D cartographic plane.
3. **Image projection:** Project 3D image data into 2D.
4. **Batch processing:** Batch process e.g. the frames of a time-lapse recording.
5. **Analysis and visualization:** Measure quantities of interest in the 2D projection, accounting for the surface’s 3D curvature.

We now describe how these steps are implemented in btc. btc comprises two tools:

1. **The** **btc** **Blender add-on** allows users to carry out tissue cartography within a graphical user interface without requiring any coding (Fig. 1C). The add-on makes Blender’s sophisticated toolset (surface sculpting, unwrapping, and annotation, Fig. 1B–B”) available for the analyses of 3D image datasets. 3D data can be visualized throughout the process, greatly facilitating, for instance, choosing a suitable cartographic projection.
2. **The** **btc** **Python library** is an open-source, cross-platform Python package available for installation on pip and allows advanced users to create custom and automatized analysis pipelines. Each pipeline step terminates in a single file of standardized type (e.g. a .obj mesh file). Due to this modular design, the tool used for each step can be easily switched out. This is essential as scientific imaging data is often highly diverse. Provided tutorial Jupyter notebooks can serve as starting points for specialized pipelines.

Comprehensive documentation (including algorithm details) for both the library and the add-on, tutorials, and a set of template analysis pipelines are available online.

### Extraction: segmentation and meshing

To extract the surface of interest, the volumetric image is first segmented, detecting either (a) the voxels that are part of the SOI or (b) the voxels that are part of the solid object whose boundary is the SOI (see examples in Fig. 3A–A”). btc provides an interface to carry out this binary segmentation using the popular machine learning software ilastik [12] and via level set algorithms [13]. Next, the resulting binary mask is converted into a surface mesh using Poisson reconstruction [14] or the marching cubes algorithm (depending on the option (a) or (b) above). Throughout btc, surfaces are represented as polygonal meshes $\left(𝒱,𝓣\right)$, where 𝒱 is a set of vertex positions, and 𝓣 is a set of polygonal faces that stitch the vertices together to form a surface. btc contains a variety of functions for mesh quality improvement (remeshing), smoothing, and processing.

Within Blender, the resulting meshes can be inspected and potential segmentation errors can be fixed using Blender’s 3D editing tools (Fig. 1B). The volumetric image data can also be loaded into Blender, allowing the user to sculpt the mesh while visualizing the image data in 3D or projected onto the mesh surface.

### Surface unwrapping and image projection

Now, the mesh can be cartographically unwrapped to a 2D plane (Fig. 1A’). This is known as *UV mapping* in the graphics community as the 2D plane coordinates are conventionally denoted u, v (versus x, y, z for the 3D coordinates). Blender possesses a powerful graphical UV editor that implements a variety of cartographic algorithms, from standard axial, spherical, and cylindrical projections to powerful, state-of-the-art algorithms like SLIM [10] which can unwrap even complex surfaces with minimal distortion. Additionally, Blender allows the user to define the location of *seams* (cartographic cuts, (Fig. 1B), manually fine-tune the results, and visualize the projection (Fig. 1B’).

The resulting UV map of a surface $\left(𝒱,𝓣\right)$ is represented in a standardized fashion as a set of 2D UV vertices in the unit UV square ${𝒱}_{\text{uv}}\subset {\left[0,1\right]}^{2}$, and a set of UV faces ${𝓣}_{\text{uv}}$ that are in one-to-one correspondence with the 3D faces. This defines how the 3D surface is mapped to the plane. The mesh and its UV map can be exported to or loaded from an .obj mesh.

Given the SOI mesh with a UV map, btc uses an interpolation algorithm to project voxel intensities from the volumetric data onto the 2D UV plane. We refer to the result as a *2D projection* (Fig. 1A’, bottom). Using the local surface normals, btc can create multi-layer projections, offset inwards or outwards by a desired distance from the SOI (analogous to peeling off the layers of an onion). The interpolation scheme is designed so that the resolution of the 2D projection does not depend on that of the mesh, i.e. a coarse, lightweight mesh can still be used for high-resolution projections. btc features a second, simpler projection algorithm that evaluates 3D image intensities at mesh vertices. The vertex-based algorithm does not require a UV map and can be recomputed rapidly, allowing the user to visualize image intensities on the mesh surface while sculpting or unwrapping it.

### Analysis and visualization

btc saves the 2D projection (and 3D positions for each pixel in the projection) both as .tif stacks for 2D visualization and analysis, as well as Blender textures for visualization on the 3D mesh. This allows not only rendering high-quality figures but also rapid, iterative improvements of the mesh and its UV map. For instance, the user can adjust the placement of cartographic seams based on the image textures, e.g. focusing on a region of particular interest. Further, Blender’s “texture paint” tools can annotate data in 3D, with results (e.g. cell labels) saved on top of 2D projections for downstream analysis (Fig. 1B”).

For quantitative analysis, btc provides a suite for measuring and correcting for cartographic distortion and mapping quantities measured in 2D back (like cell tracks or out-lines)back to 3D. For instance, these tools can correctly compute the area of a cell in a 2D projection, even if the unwrapping introduces local area distortion. All required quantities can be robustly computed based on the triangular mesh structure [15].

We also provide a simple implementation of vector calculus on curved surfaces, for instance, to analyze morphogenetic tissue flows obtained from particle image velocimetry (PIV) or cell tracking in 2D projections [4, 2]. In brief, vector and tensor fields on a surface are represented by their Cartesian 3D coordinates and then separated into tangential and normal components. Using standard finite-element operators on the mesh, gradients can be calculated component-wise and combined to form the curved-surface generalizations of familiar operators like div, grad, and curl [16]. This approach is more accessible to non-experts than elegant discrete differential geometry methods [15].

### Batch processing and dynamic datasets

The workflow described so far applies to a single surface of interest. Often, however, we are interested in dynamic datasets (movies), or multiple recordings of similarly shaped objects (e.g. multiple *Drosophila* blastoderm stained with different markers[17]). In btc, time-lapse datasets are represented frame-by-frame (one surface mesh and volumetric image file per timepoint). Such collections of SOIs can be batch-processed via the btc add-on or Python library.

It is highly desirable to use “the same” UV map for each time point, both to facilitate comparison of the 2D projection across time points or samples and because it would be cumbersome to manually define a UV map each time. There are two possible strategies.

The first possibility is to compute a UV map for each mesh individually, but using a consistent algorithmic procedure. This approach works well for simple shapes that can be unwrapped by axis, cylindrical, or spherical projections which can be batch-computed in Blender. However, for more complex shapes (in particular, if unwrapping requires seams), choosing or designing a suitable algorithm can require significant computer vision expertise and, crucially, cannot be done graphically and interactively.

**Figure 2:**
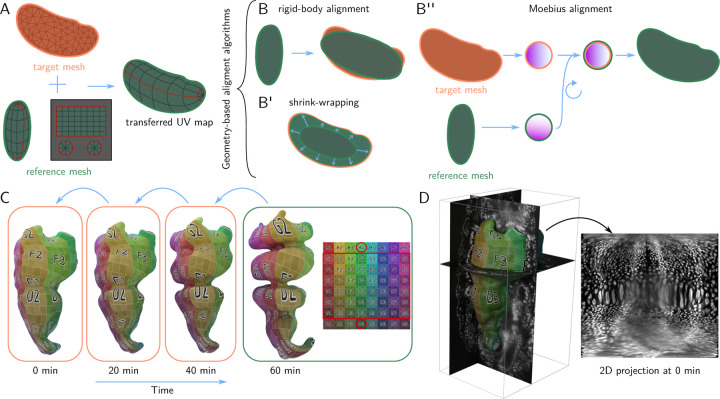
Batch-processing and dynamic data via UV surface-surface alignment. **A** Transferring a UV map to the orange target mesh from the green reference mesh via surface-to-surface alignment: the reference mesh is deformed to match the shape of the target mesh while preserving its UV map. **B-B”** Surface-to-surface alignment algorithms: rigid-body alignment, shrink-wrapping (smoothed closest-point projection), and Moebius alignment (see text). All algorithms are based on the shape of the surface only. **C** UV transfer for a highly dynamic biological surface. Mesh of the embryonic *Drosophila* midgut extracted from live deep-tissue *in-toto* imaging (data from [2]). The complex shape of the final timepoint is unwrapped in Blender and the UV map is iteratively transferred to all other timepoints via shrink-wrapping. **D** The extracted surface at timepoint 0 together with 3D data (fluorescently marker nuclei) and 2D projection.

Instead, in btc, the user defines a UV map for a selected *reference mesh* – e.g. the first or last frame of a movie, or an “idealized” version of the imaged shapes – using Blender’s graphical UV editor. The reference mesh is then algorithmically mapped onto the meshes of the remaining time points (the *target meshes*), a process that we refer to as *surface-to-surface alignment* (Fig. 2A). This allows *transferring the UV map* from the reference mesh to all other meshes and hence creates coherent 2D projections across time points or samples. In particular, the layout of the 2D projection in the UV square does not change. For the case of multiple samples, surface-to-surface alignment allows combining recordings of e.g. different markers into a single “atlas” [17].

Surface-to-surface registration has received significant attention in the computer graphics community. In btc, we implement three algorithms, all based purely on surface geometry (i.e. they make no use of the 3D image data):

1. **Rigid-body alignment.** The reference mesh is translated, rotated, and scaled to match the target mesh (Fig. 2B).
2. **Closest-point projection (shrink-wrapping).** The reference mesh is first rigidly aligned to the target, and then each vertex of the reference mesh is projected to the closest point on the target surface (Fig. 2B’). This operation is combined with smoothing to remove “creases”.
3. **Moebius alignment** [18]. Both reference and target mesh are mapped to a reference shape (a disk, a punctured disk, or a sphere, depending on the surface topology) in a way that preserves triangle angles. Mathematically, this *conformal map*^1^ to the reference shape is guaranteed to be almost unique, up to a small set of *Moebius transformations* (e.g. rotations) which are chosen to best align the geometry of the reference and target mesh (Fig. 2B”).

For dynamic datasets, surface-to-surface registration can done iteratively (i.e. first map reference to the first frame, then the result to the second frame, etc.), significantly reducing its difficulty (Fig. 2C).

Thanks to btc’s extensible Python interface, external libraries such as PyFM (implementing so-called “functional maps” [21]) can also be used. The above algorithms are fully automatic and do not require the user to specify point-to-point correspondences between reference and target mesh, which is time-consuming and can introduce bias in the absence of clear shapes enabling interest point placement. The Moebius algorithm is highly robust and will find a surface-to-surface map even between two strongly deformed meshes [18].

## Results

We now showcase btc on a range of example datasets.

### Dynamic shapes

We validated btc’s approach to time-lapse data on an example of a highly dynamic surface, the embryonic *Drosophila* midgut (Fig. 2C–D, data from Ref. [2]). We find that it is best to choose the time point with the most complicated shape (here, the final one), as the reference mesh. The reference mesh is unwrapped in Blender, along a user-defined seam that follows the long axis of the organ. The UV map is then iteratively transferred to the remaining transferred using the closest-point projection algorithm (Fig. 2C), allowing computing projections across all time points (Fig. 2D). The algorithm faithfully transfers the UV map across time points, with only minor degradation close to mesh seams.

### Diverse and complex shapes

**Figure 3:**
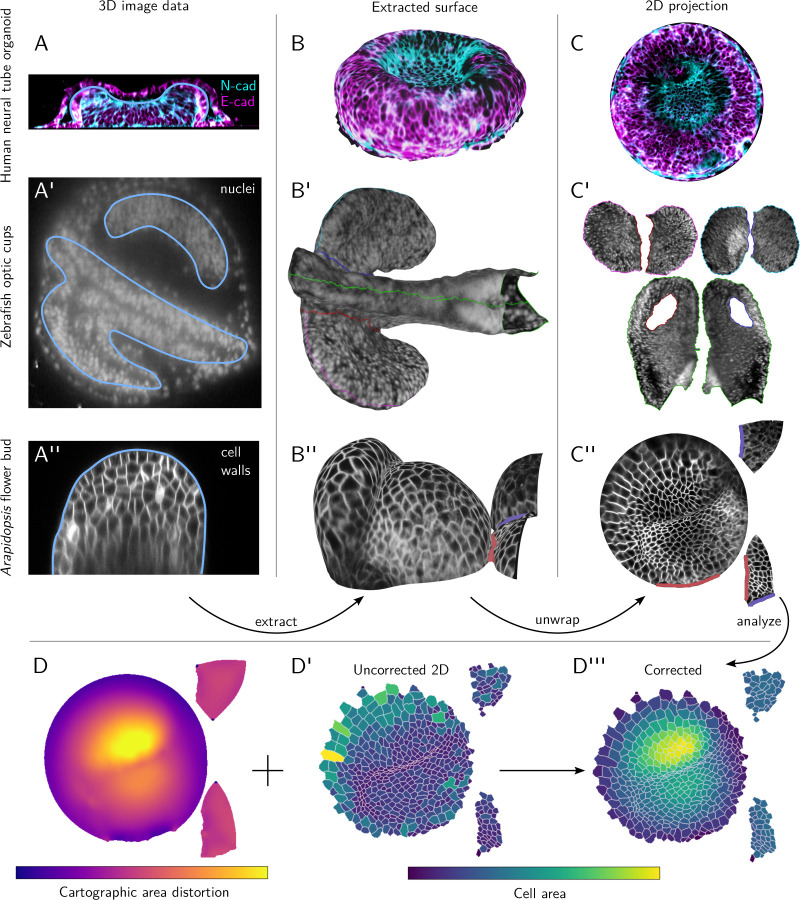
btc can process complex and diverse shapes across many biological contexts. **A-A”** Cross-sections of 3D image data with extracted surface contoured in light blue (boundary of 3D segmentation). (A) Confocal *z*-stack of a human neural tube organoid [22], stained for neural- and epithelial cadherin. Sideview, substrate at bottom. The extracted organoid develops within a lumen, visible outside the blue contour. (A’) Lightsheet recording of a 16hpf zebrafish embryo, with fluorescently marked nuclei. Anterior left, ventral view. (A”) Confocal *z*-stack of *Arabidopsis thaliana* flower buds with a membrane marker. **B-B”** 3D renderings of the extracted surfaces, with seams of the UV map marked in color. **C-C”** Corresponding 2D projections. **D-D”** Quantitative analysis using cartographic projections. (D) Area distortion of UV map from C”. (D’-D”) Segmentation of 2D projected *Arabidopsis thaliana* data, colored by uncorrected 2D cell areas (D’) and cartographically corrected, 3D cell areas (D”).

Next, we demonstrate the applicability of btc to complex and diverse shapes from a range of biological contexts (Fig. 3, data sources described in Materials & Methods).

Our first example is a confocal *z*-stack of a human neural tube organoid grown on a micropatterned substrate [22] (Fig. 3A–C). Even for this relatively simple shape, tissue cartography offers clear advantages over a maximum *z*-projection, as well as height-map-based approaches like LocalZProjector [8], both of which are unable to follow the curved apical surface of the organoid due to overhangs. Note also that btc supports arbitrary multichannel data (here, neural and epithelial cadherin stainings).

Next, we extracted the anterior neural tube and developing optic vesicles [23] from an *in-toto* light-sheet recording of a 16hpf zebrafish embryo (Fig. 3A’–C’). This complex shape is beyond the reach of the ImSAnE or TubULAR software, but can be easily unwrapped along seams following morphological landmarks, selected graphically within Blender (Fig. 3B’).

Our final example are *Arapidospis thaliana* flower buds (Fig. 3A”–B”). The cartographic projection Fig. 3C” can be used for quantitative analysis (Fig. 3D–D”), in this case cell segmentation. The btc Python library can compute and correct for cartographic distortion, for example when quantifying cell areas, ensuring faithful 3D measurements in a 2D projection.

## Discussion

Here, we presented blender_tissue_cartography (btc), a software suite for analyzing dynamic 3D microscopy data via the 3D editor Blender. Tissue cartography extracts and cartographically projects surface-like structures from volumetric data, reducing 3D to 2D data analysis, and enabling quantitative insights into morphogenetic dynamics during animal and plant development. Thanks to the graphical user interface, suitable cartographic projections can be iteratively designed for new and complicated geometries. Our pipeline for dynamic movies preserves the ability of the user to graphically define their UV map and is applicable without modification across a wide range of datasets. We have demonstrated btc on a variety of static and dynamic datasets with complex geometries from four different model organisms. In our experience, btc has proven user-friendly and easy to learn, with second-year undergraduates and scientists with no computer programming experience able to analyze the data of their interest without additional instruction. We hope btc will make the analysis of dynamic 3D surfaces accessible to a broad audience and thus further our understanding of morphogenesis.

## Materials and methods

### btc Python library

The btc Python library is built using the standard Python scientific computing software stack [24, 25, 26] and uses a lightweight mesh data representation inspired by the .obj mesh file format. The geometry-processing library igl is used as the main geometry backend [27]. MeshLab [28] is used for certain advanced (re)meshing operations. The source code is available on GitHub. The Blender add-on is based on the btc library code with adaptions to interface with the Blender API.

### Data sources

The *Drosophila* blastoderm in Fig. 1A shows a multiview light-sheet microscope recording of a *+;+;H2A:RFP* embryo at stage 6 from Ref. [17]. Fig. 2C–D shows the embryonic *Drosophila* midgut extracted from a multiview lightsheet microscope recording of a *Hand:GFP;+;Hist:GFP* embryo at stage 15 from Ref. [2]. Fig. 3A–C shows a confocal microscope recording of a human neural tube organoid stained for N-cadherin and E-cadherin at 48 hours post BMP addition (organoid protocol initiation) from Ref. [22]. Fig. 3A”–C” shows a confocal recording of *Arabidposis thaliana* flower buds with plasma membrane marker *pUBQ10::29-1-GFP* [29], shared Dr. An Yan. Permission to display the data was obtained from the respective authors of the respective publications. The zebrafish data in Fig. 3A’–C’ was recorded for this manuscript, see next section.

### Zebrafish microscopy

Adult zebrafish of the AB/TU background were used for experiments and housed and bred using standard conditions [30]. All experiments were performed with institutional approval from the University of California, Santa Barbara. Zebrafish embryos were mounted in agarose and imaged in a custom multi-view light-sheet microscope described previously [31], and multi-view data was processed as described previously [31, 32] to obtain volumetric image data. Cell nuclei were visualized using the *Tg(h2afva:h2afva-GFP)* reporter line [33].

### Data processing

Data was processed following the pipeline described in the main text methods section. In brief, all volumetric datasets were segmented using ilastik with additional post-processing via level set methods for the zebrafish dataset. The volumetric segmentations were converted to meshes using the btc Blender add-on, and remeshed for improved mesh quality where necessary using either Blender or MeshLab. For the embryonic *Drosophila* midgut data, we used surface meshes generated in Ref. [2]. UV maps were generated using the “Minimum Stretch” and “Angle Based Flattening” algorithms in Blender [10] after graphical seam placement. To obtain a cylinder-like projection for the embryonic midgut data, we used the “Pin” and “Align” tools of the UV editor to ensure straight seams. All 2D projections and 3D renderings were created in Blender, using the btc add-on.

## Footnotes

1 Technically, btc can use least-squares conformal [19] or harmonic maps [20].
